# EPA/DHA and Vitamin A Supplementation Improves Spatial Memory and Alleviates the Age-related Decrease in Hippocampal RXRγ and Kinase Expression in Rats

**DOI:** 10.3389/fnagi.2016.00103

**Published:** 2016-05-09

**Authors:** Anne Létondor, Benjamin Buaud, Carole Vaysse, Emmanuel Richard, Sophie Layé, Véronique Pallet, Serge Alfos

**Affiliations:** ^1^Université de Bordeaux, Nutrition et Neurobiologie Intégrée, UMR 1286Bordeaux, France; ^2^INRA, Nutrition et Neurobiologie Intégrée, UMR 1286Bordeaux, France; ^3^Bordeaux INP, Nutrition et Neurobiologie Intégrée, UMR 1286Bordeaux, France; ^4^ITERG, Institut des Corps GrasPessac, France; ^5^INSERM, Biothérapie des Maladies Génétiques Inflammatoires et Cancers, U1035Bordeaux, France

**Keywords:** n-3 long-chain PUFA, vitamin A, spatial memory, hippocampus, kinases, retinoid receptors

## Abstract

Studies suggest that eicosapentaenoic acid (EPA), docosahexaenoic acid (DHA), and vitamin A are critical to delay aged-related cognitive decline. These nutrients regulate gene expression in the brain by binding to nuclear receptors such as the retinoid X receptors (RXRs) and the retinoic acid receptors (RARs). Moreover, EPA/DHA and retinoids activate notably kinase signaling pathways such as AKT or MAPK, which includes ERK1/2. This suggests that these nutrients may modulate brain function in a similar way. Therefore, we investigated in middle-aged rats the behavioral and molecular effects of supplementations with EPA/DHA and vitamin A alone or combined. 18-month-old rats exhibited reference and working memory deficits in the Morris water maze, associated with a decrease in serum vitamin A and hippocampal EPA/DHA contents. RARα, RXRβ, and RXRγ mRNA expression and CAMKII, AKT, ERK1/2 expression were decreased in the hippocampus of middle-aged rats. A combined EPA/DHA and vitamin A supplementation had a beneficial additive effect on reference memory but not in working memory in middle-aged rats, associated with an alleviation of the age-related decrease in RXRγ, CAMKII, AKT, and ERK1 expression in the hippocampus. This study provides a new combined nutritional strategy to delay brain aging.

## Introduction

Brain aging is associated with multiple morphological and biochemical changes leading to cognitive decline such as learning and memory impairments (Lister and Barnes, 2009). Nutrition is one of the multiple environmental factors which can contribute to successful aging and may modulate mental health (Parletta et al., 2013). Among the dietary nutrients most closely associated with optimal brain functioning, two n-3 LC-PUFAs, namely DHA and EPA, are particularly involved in the maintenance of cognitive functions during aging (Su, 2010; Joffre et al., 2014). The ability of these n-3 LC-PUFAs to modulate brain functions depends on their accretion level in the brain, closely related to the dietary intake and age. Indeed, aging is associated with a decrease in brain n-3 LC-PUFA contents (Labrousse et al., 2012). More specifically, an age-related decrease in DHA content in the hippocampus has been observed (Favrelière et al., 2003; Dyall et al., 2007). Moreover, we have recently shown that an EPA-DHA supplementation can reverse the age-related decrease in brain DHA content (Letondor et al., 2014). Such a supplementation has already been shown to improve memory performance in aged animals (for review, see Alfos, 2014).

Besides, brain aging is associated with an hypoactivation of the RA signaling pathway which could be involved in age-related memory impairments (Mingaud et al., 2008; Dyall et al., 2010; Bonhomme et al., 2014). RA, the active metabolite of vitamin A, plays a key role in the regulation of synaptic plasticity and in learning and memory in adults (Lane and Bailey, 2005). Indeed, it has been shown that the administration of RA improves reference and relational memory performance of aged animals by restoring the expression of retinoid nuclear receptors (RAR and RXR) and of synaptic plasticity markers in the hippocampus (Etchamendy et al., 2001). Similarly, Dyall et al. (2010) have shown that the age-related decrease in RARα, RXRα, and RXRβ protein levels in the rat forebrain and in the CA1 and dentate gyrus of the hippocampus are reversed by a 12 weeks EPA/DHA supplementation. Moreover, we have recently shown that a mid-life vitamin A supplementation during 4 months prevents spatial memory decline in 17-month-old rats and improves the dendritic arborisation of newborn immature neurons by inducing an increase in the intracellular availability of RA (Touyarot et al., 2013).

Retinoids and n-3 LC-PUFAs may modulate cerebral plasticity and memory by regulating gene expression through nuclear receptors that function as ligand-controlled transcription factors (Lane and Bailey, 2005; Su, 2010). Indeed, DHA and RA can bind to nuclear receptors, such as the PPARs, the RARs, and the RXRs (Evans and Mangelsdorf, 2014). Several studies highlighted multiple levels of interactions between the fatty acid and the retinoid signaling pathways. On the one hand, it has been shown that RXR is the obligatory heterodimerization partner of RARs and PPARs, suggesting that RXRs play a key role in both retinoid- and n-3 PUFA-mediated signaling pathways (van Neerven et al., 2008). On the other hand, *in vitro* studies have shown that fatty acids and particularly DHA can bind and activate RXRs (de Urquiza et al., 2000; Lengqvist et al., 2004) and that RA can bind to the PPARs (Shaw et al., 2003; Schug et al., 2007), implying interactions at the nuclear level between DHA and RA for binding to their receptors.

Moreover, RA and n-3 LC-PUFAs have additional extra-nuclear and non-transcriptional effects that activate kinase signaling pathways such as, AKT or the MAPK, which includes ERK1/2, thus influencing gene expression through phosphorylation processes (Masia et al., 2007; Rao et al., 2007; Al Tanoury et al., 2013). These signaling pathways are involved in the modulation of cerebral plasticity and thus in learning and memory processes (Giese and Mizuno, 2013). It has been shown that the ERK2 mRNA expression is impaired in the rat hippocampus during aging (Simonyi et al., 2003). A disruption of the AKT signaling pathway was also recently highlighted in a mouse model of accelerated-senescence (Armbrecht et al., 2014). Other kinases such as the CAMKII involved in synaptic plasticity (Ma et al., 2015) seem to be modulated by both n-3 PUFAs and RA. Indeed, although the transcriptional regulation of CAMKII depends on retinoids (Chen and Kelly, 1996), it has been shown that DHA treatment normalizes the CAMKII expression in the hippocampus of rats after a traumatic brain injury (Wu et al., 2011).

Altogether these data indicate that there is a close relationship between the n-3 LC-PUFA and the retinoid signaling pathways with both intra- and extra-nuclear interactions, suggesting that these nutrients may act together to modulate synaptic plasticity processes and memory altered during aging.

The present study therefore evaluates in middle-aged rats the potential synergetic behavioral and neurobiological effects of nutritional supplementation with EPA-DHA and vitamin A. For this purpose, spatial reference memory and working memory were assessed in the Morris water maze. To specify the molecular mechanisms mediated by the dietary supplementations on memory processes, we measured mRNA expression of RXRs and RARs and kinases CAMKII, AKT, and ERK1/2 and their protein levels in the hippocampus.

## Materials and Methods

### Animals and Diets

The study was conducted according to the INRA Quality Reference System and to the directive 2010/63/UE of the European Parliament and of the Council on the protection of animals used for scientific purposes. The protocols were approved by the French Ministry for Higher Education and Research and the Animal Care and Use Committee of Bordeaux (n°. 5012051-A).

3-week-old and 13-month-old male Wistar rats were purchased from Janvier (France) and maintained under standard housing conditions in a temperature- (22 ± 1°C) and humidity-controlled room (40%) with a 12-h light/dark cycle. All the animals were fed and given water *ad libitum*. After 1 week of acclimatization to the housing conditions with a standard chow, the 13-month-old rats were randomly divided in four groups (*n* = 9–10 per group): the first group received a control diet (middle-aged control group), the second group received an EPA/DHA-enriched diet (middle-aged EPA/DHA group), the third received a vitamin A-supplemented diet (middle-aged Vit A group) and the fourth received an EPA/DHA + vitamin A-enriched diet (middle-aged EPA/DHA + Vit A group). The 3-week-old rats (*n* = 10) received exclusively the control diet (adult control group). All the diets were given for 21 weeks therefore adult rats were 6-month-old and middle-aged rats were 18-month-old at the end of the experiment.

The control and vitamin A diets were free from LC-PUFA and consisted of a mix of peanut, rapeseed and sunflower oils (60/25/15, by weight) added to a fat-free diet containing a standard amount of 5 IU/g diet of vitamin A (control group) or enriched with 45 IU/g diet of vitamin A (Vit A group; UPAE-INRA Jouy-en-Josas, France). The EPA/DHA supplemented diet was a mix of fish, rapeseed and sunflower oils (50/20/30, by weight) added to the fat-free diet (EPA/DHA group) or the fat-free diet enriched in vitamin A (EPA/DHA + Vit A group). The composition of the different diets is detailed in **Table 1**. The fatty acid composition of the diets was assessed by gas chromatography as previously described (Buaud et al., 2010). Rats had a maximal daily intake of 395 mg/kg body weight EPA and 403 mg/kg body weight DHA. Fish oil was aliquoted in glass bottles under nitrogen and stored at 4°C for 21 weeks. The diets were freshly prepared every 2 days and stored at 4°C until their use. Food was changed daily between 5:00 and 7:00 p.m. and any left-over food was discarded. At the end of the 21-weeks feeding period, the adult rats (6-month-old) and the middle-aged rats (18-month-old) were anesthetized with isoflurane and rapidly decapitated. Blood was collected from the sectioned jugular vein. Brains were quickly removed, the whole hippocampus was dissected bilaterally and stored at -80°C until further analysis.

**Table 1:** Composition of experimental diets.

	Control	Vit A	EPA/DHA	EPA/DHA + Vit A
**Ingredients *(% of total provided energy)***				
Lipids	11.2	11.2	11.2	11.2
Saturated	1.6	1.6	1.0	1.0
Monounsaturated	6.9	6.9	3.4	3.4
Polyunsaturated	2.7	2.7	6.8	6.8
Proteins	18.0	18.0	18.0	18.0
Carbohydrates	69.8	69.8	69.8	69.8
Energy (kJ/g of diet)	16.7	16.7	16.7	16.7
Vitamin A (IU/g of diet)	5.0	45	5.0	45
**Fatty acid composition *(g/100 g of diet)***				
16:0	0.4	0.4	0.2	0.2
18:0	0.2	0.2	0.2	0.2
18:1n-9	3.0	3.0	1.6	1.6
18:2n-6	1.1	1.1	1.2	1.2
20:4n-6	0.0	0.0	0.1	0.1
22:5n-6	0.0	0.0	0.0	0.0
18:3n-3	0.1	0.1	0.1	0.1
20:5n-3 (EPA)	0.0	0.0	0.7	0.7
22:5n-3	0.0	0.0	0.1	0.1
22:6n-3 (DHA)	0.0	0.0	0.7	0.7
Total SFAs	0.6	0.6	0.4	0.4
Total MUFAs	3.0	3.0	1.6	1.6
Total PUFAs	1.2	1.2	2.9	2.9
Total n-6 PUFAs	1.1	1.1	1.3	1.3
Total n-3 PUFAs	0.1	0.1	1.6	1.6
18:2n-6/18:3n-3	11	11	12	12
n-6 PUFA/n-3 PUFA	11	11	0.8	0.8

*MUFAs, monounsaturated fatty acids; PUFAs, polyunsaturated fatty acids; SFAs, saturated fatty acids.*

### Behavioral Testing

Reference memory and working memory were tested in a Morris water maze (180 cm diameter, 60 cm high) filled with water (21–22°C) made opaque with non-toxic white paint. Before the learning phase, animals were habituated to the pool without any platform 60 s/day for 2 days. The principle of this test is based on the capacity to memorize and to develop a spatial map of the extra-maze cues to find an escape platform hidden 2 cm below the surface of the water. For each trial, the distance swum, the speed and the latency to reach the platform were measured with a computerized tracking system (Videotrack, Viewpoint, Lyon, France).

#### Spatial Learning and Reference Memory (Place Version)

Spatial reference memory was evaluated according to the protocol of Bonnet et al. (2008) modified as follow. Rats were trained for four consecutive trials a day (90 s with an inter-trial of 30 s, starting from different points in a randomized order every day) for nine consecutive days (learning phase). The distance swum to reach the platform should decrease over testing sessions (days) as the rats learn the location of the platform. 24 h after the last training day (day 10), the probe test was performed by placing the rats for 60 s in the pool without the platform. Reference memory was evaluated by measuring the percentage of time spent in the quadrant where the platform was during the learning phase (target quadrant).

#### Working Memory (Matching-to-Place Version)

Working memory was evaluated according to the protocol of Wainwright et al. (1999) modified as follow. 48 h after the probe test, rats were tested in the matching-to-place version of the Morris water maze. In each testing session, the rats received a pair of trials in which the start position was varied pseudo-randomly, but the platform remained at the same place. However, in contrast to the reference memory task, the location of the platform is changed everyday for 6 days. On the first 3 days, animals were tested with an inter-trial interval (ITI) of 30 s and an ITI of 2 min was applied on the last 3 days. In this version of the task, each of the testing session can be considered as a separate “problem” in which the first trial is a search trial and the second trial is a test trial which highlights the ability to remember the immediately preceding location of the platform in the first trial.

#### Cued Learning Version

24 h after the end of the working memory phase, rats were tested (one session with four trials) to find a cued visible platform. Differences in performance on this task would be indicative of potential alterations in the sensory, motor or motivational attributes of the animals.

### Measurement of Serum Retinol Concentration

Blood collected during the sacrifice was immediately spun at 1,500 g for 15 min. The supernatant was removed and snap frozen on dry ice. Serum retinol was assessed by HPLC according to the method previously described by Leclercq and Bourgeay-Causse (1981).

### Hippocampal Phosphatidylcholine and Phosphatidylethanolamine Analysis

Total lipids from the other half of the hippocampi were extracted and PC and phosphatidylethanolamine (PE) were isolated from other lipid classes by thin layer chromatography as previously described (Letondor et al., 2014). PC and PE total fatty acids were transmethylated and analyzed in a blinded fashion by gas chromatography as previously described (Letondor et al., 2014). Results are expressed as percentage of PC and PE total fatty acids.

### mRNA Expression Analysis by Real-time PCR

The other half of the dissected hippocampi was homogenized in 1 ml of Trizol reagent (Invitrogen, France) and total RNAs were extracted according to the manufacturer’s instructions. The organic phase containing proteins was stored at -20°C for subsequent Western blot analysis. The concentration of the purified RNA was measured by spectrophotometry at 260 nm using a Nanodrop ND-1000 (Labtech, France). The integrity of RNA samples was assessed using the RNA 6000 Nano LabChip kit in combination with the 2100 Bioanalyzer (Agilent Technologies, France). RNAs were reverse transcribed in complementary DNAs using Improm II reverse transcriptase (Promega, France) according to the manufacturer’s instructions. Real-time PCR were run on a LigthCycler 480 thermal cycler using the SYBR Green I Master kit (Roche Diagnostics, Mannheim, Germany) as previously described (Bonnet et al., 2008). The forward and reverse primer sequences used are shown in **Table 2**. The specificity and identity of the amplified products were verified by the melting curve analysis showing a single melting peak after amplification and by sequencing the amplified PCR products using the Big Dye Terminator v1.1 and an ABI3130 sequencer (Applied Biosystems). Data analysis was performed using the Roche’s E-method of relative quantification, which uses standard curve derived efficiencies, of the LightCycler 480 1.5 version software. In this study we used the β2-microglobulin (BMG) housekeeping gene as the reference gene since its expression level was unaffected by our experimental conditions. The results are expressed as the target/reference ratio divided by the target/reference ratio of the calibrator.

**Table 2:** Primers used for quantitative RT-PCR.

Gene name	Nucleotide sequence	Product length (bp)
BMG	F: 5′-GCCCAACTTCCTCAACTGCTACG-3′	180
	R: 5′-GCATATACATCGGTCTCGGTGGG-3′	
RARα	F: 5′-GCCTCGATTCTACAAGCCTTGC-3′	107
	R: 5′-GGATACTGCGTCGGAAGAAGC-3′	
RARβ	F: 5′-CAGCTGGGTAAATACACCACGAA-3′	227
	R: 5′-GGGGTATACCTGGTACAAATTCTGA-3′	
RARγ	F: 5′-GCCCTAAGGCTTTATGCCCGG-3′	104
	R: 5′-GCTCCCTTGGTGCTGATGCCC-3′	
RXRα	F: 5′-GCTGGTGTCGAAGATGCGTGAC-3′	171
	R:5′-GGGTACTTGTGTTTGCAGTACG-3′	
RXRβ	F: 5′-TGGGAACAGGGAGAATGTGG-3′	129
	R: 5′-CTGGAAAGCGACTTTATGTGCAAG-3′	
RXRγ	F: 5′-GGAAAGACCTCATCTACACG-3′	123
	R: 5′-CAGCTTCCCTCTTCATGCCC-3′	
ERK1	F: 5′-TCCCCTTGACCTGAGTGATGAG-3′	102
	R: 5′-CCATTCCAGAACCGTCTACCAGA-3′	
ERK2	F: 5′-CGTCTCAGCTTACCCACTCTTGA-3′	109
	R: 5′-TGCAGGAGAACTCTCTGGACTG-3′	
CREB	F: 5′-GTTCAAGCCCAGCCACAGATT-3′	84
	R: 5′-GGTTACAGTGGGAGCAGATGAC-3′	
CAMKII	F: 5′-TGCACAGACAGGAGACCGTGGAC-3′	122
	R: 5′-GTTTCCTCCACTCTTCCCTCCGG-3′	
AKT	F: 5′-TGAGCGCGTGTTTTCAGAGG -3′	131
	R: 5′-CCTTGTCCAGCATGAGGTTCTC -3′	

*Sequences are shown for forward (F) and reverse (R) primers.*

### Western Blot Analysis

Total proteins from half hippocampi were extracted from the Trizol fraction previously recovered from the RNA extraction step according to the manufacturer’s instructions slightly modified by Simoes et al. (2013). Briefly, the tube containing the organic phase was centrifuged at 12,000 *g* for 15 min at 4°C, and the remaining supernatant was removed and discarded. DNA was precipitated by addition of 100% ethanol and centrifuged at 2,000 *g* for 5 min at 4°C. Proteins in the phenol-ethanol supernatant were precipitated by addition of isopropanol and centrifuged at 12,000 *g* for 10 min at 4°C. The protein pellet was next washed three times with 0.3 M guanidine hydrochloride in 95% ethanol. After the final wash and centrifugation at 7,500 *g* for 5 min at 4°C, proteins were precipitated by addition of 100% ethanol followed by a final centrifugation at 7,500 *g* for 5 min at 4°C. The protein pellet was solubilized by sonication in a 1:1 solution of 1% SDS and 8 M urea in Tris-HCl 1 M, pH 8.0. The samples were centrifuged at 3,000 *g* for 10 min at 4°C, to sediment insoluble material and the supernatant containing the solubilized proteins was stored at -80°C. Protein concentration was determined using the MicroBC Assay protein quantitation kit. Western blot analysis was performed as previously described by Boucheron et al. (2006) with slight modifications. Briefly, aliquots containing 40 μg of total proteins were resolved on 10% sodium dodecyl sulfate-polyacrylamide gel and transferred to immobilon polyvinyllidene difluoride membranes (Millipore, Billerica, MA, USA). Blots were incubated overnight at 4°C with primary rabbit anti-actin (diluted 1:5000, Sigma, France), anti-AKT, anti-phospho-AKT, anti p44/42 MAPK (ERK1/2) or anti-phospho-p44/42 MAPK (phospho-ERK1/2), diluted 1:1000 (Cell Signaling Technology, Danvers, MA, USA). After washing, the blots were incubated with appropriated horseradish peroxidase-conjugated secondary antibodies (Jackson Immunoresearch, Westgrove, PA, USA). Following several washings, the bands were developed using the Western Lightning Chemiluminescence Reagent Plus (PerkinElmer Life Science, Waltham, MA, USA) and quantified by measuring chemiluminescence with an image analysis system (Syngene, Frederick, MD, USA). The relative levels of proteins in middle-aged control and supplemented rats were expressed as a percentage of the same proteins in adult rats. The constant level of actin was verified and found to be identical in all groups (**Figure 6A**).

### Statistical Analysis

Results are expressed as mean ± standard error of the mean (mean ± SEM). Statistical analyses were performed with StatView 5.0 software. For reference memory, data were analyzed by a one-way ANOVA or repeated measures ANOVA (for learning phase) followed by a Fischer PLSD *post hoc* test and a Student’s *t*-test to compare with chance level. For working memory data were analyzed by a two-way (group × trial) ANOVA followed by a paired Student’s *t*-test to compare trials within groups. *P* values < 0.05 were considered to be statistically significant.

## Results

### Spatial Learning and Reference Memory

**FIGURE 1:**
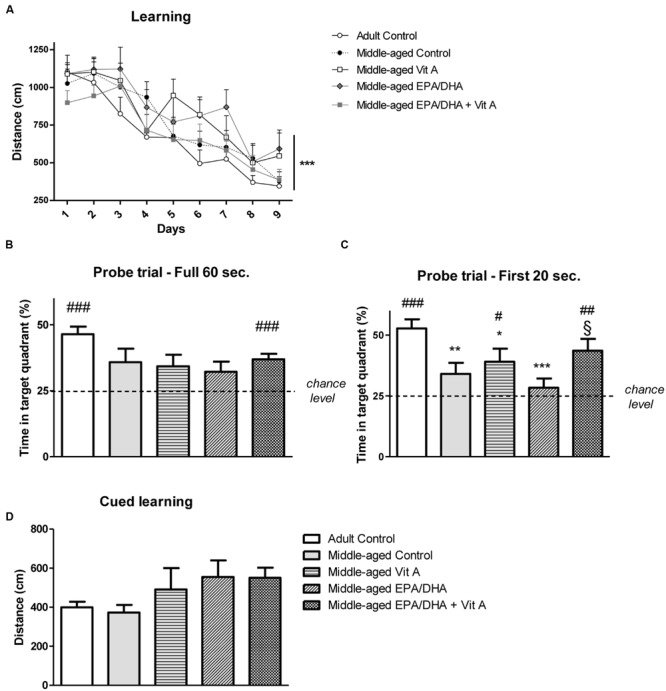
**Effect of age and diet (EPA/DHA, Vit A, or EPA/DHA + Vit A) on spatial learning, reference memory and cued learning in the Morris water maze.** Figures show **(A)** the mean distance swum during acquisition to reach a submerged platform located at the same position over 9 days with four trials per day; **(B,C)** Percentage of time spent in the target quadrant with the platform removed in the probe test over **(B)** 60 s or **(C)** 20 s; **(D)** the mean distance swum during the cued learning. Values are mean ± SEM, *n* = 8–10 rats per group. Data were analyzed by **(A)** repeated measures ANOVA or **(B–D)** one-way ANOVA followed by the Fischer PLSD *post hoc* test. Signs indicate values different from **(A)** day 1 or **(B,C)** from Adult control: ^∗^*p* < 0.05; ^∗∗^*p* < 0.01; ^∗∗∗^*p* < 0.001 and from middle-aged EPA/DHA: §*p* < 0.05; or compared with chance level by a Student’s one group *t*-test: #*p* < 0.05; ##*p* < 0.01; ###*p* < 0.001.

During the learning phase, rats were trained in the Morris water maze over 9 days with four trials per day to find a submerged platform. The swimming speed during this phase was significantly different between middle-aged and adult rats (adult control: 22.3 ± 0.3 cm/s, middle-aged control: 18.6 ± 0.4 cm/s, middle-aged Vit A: 20.6 ± 0.4 cm/s, middle-aged EPA/DHA: 19.7 ± 0.4 cm/s, middle-aged EPA/DHA + Vit A: 19.6 ± 0.4 cm/s; *F*_(4,38)_ = 3.87; *p* < 0.01), therefore we analyzed the mean distance swum to reach the platform over the 9 days of training (**Figure 1A**). The distance to reach the escape platform decreased along the days with a main effect of days (*F*_(8,304)_ = 35.863; *p* < 0.0001), meaning that all rats learned this task. However, we did not observe any group effect on learning (*F*_(4,38)_ = 1.310; *p* = 0.284).

During the probe test, we measured the percentage of time spent in the target quadrant from where the platform was removed. The analysis of the probe test over the 60 s did not reveal any group effect (adult control: 46.4 ± 2.8%, middle-aged control: 35.8 ± 5.1%, middle-aged Vit A: 34.2 ± 4.4%, middle-aged EPA/DHA: 32.2 ± 3.8%, middle-aged EPA/DHA + Vit A: 36.9 ± 2.2%; *F*_(4,38)_ = 2.25; *p* < 0.08; **Figure 1B**). However, only adult control rats and middle-aged rats supplemented with EPA/DHA + Vit A spent significantly more than 25% (chance level) of the total time in the target quadrant (*t* = 7.472, *p* < 0.0001 and *t* = 5.501, *p* < 0.001, respectively). Then, analyzing more precisely the first 20 s (**Figure 1C**) which is more relevant to avoid a possible motivational extinction (Blokland et al., 2004), a significant group effect was observed on the time spent in the target quadrant (adult control: 52.7 ± 3.8%, middle-aged control: 34 ± 4.6%, middle-aged Vit A: 39 ± 5.4%, middle-aged EPA/DHA: 23.3 ± 4.8%, middle-aged EPA/DHA + Vit A: 43.6 ± 4.8%; *F*_(4,38)_ = 4.54; *p* < 0.01). Indeed, middle-aged control and supplemented rats with only EPA/DHA or vitamin A exhibited lower memory performance than the adult control rats. In contrast, the performance of the EPA/DHA + Vit A supplemented rats was not different from the adult control rats and even better than that of the group supplemented only with EPA/DHA (*p* = 0.026). However, the performance of the rats receiving the combined supplementation was not significantly improved over that receiving vitamin A alone (*p* = 0.5). When comparing the time spent in the target quadrant over the first 20 s with the chance level, the analysis revealed that only three groups of rats: adult control (*p* < 0.0001), middle-aged Vit A (*p* < 0.05) and middle-aged EPA/DHA + Vit A (*p* < 0.01) spent more time in the target quadrant, suggesting that only these groups remembered the location of the platform.

### Spatial Working Memory

**FIGURE 2:**
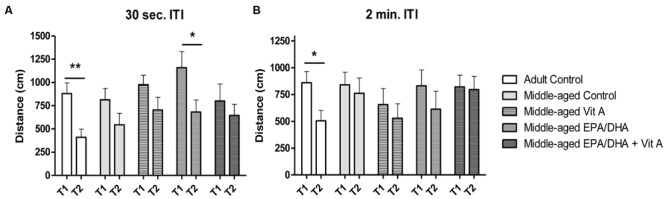
**Effect of age and diet (EPA/DHA, Vit A, or EPA/DHA + Vit A) on spatial working memory (matching-to-place version) in the Morris water maze.** Figures show **(A)** the mean distance swum to reach a submerged platform over 3 days in the first trial (T1) followed by a second trial (T2) with an inter-trial interval (ITI) of 30 s; **(B)** the mean distance swum to reach a submerged platform over 3 days in the T1 and T2 with an ITI of 2 min. The platform was moved to a different location each day. Values are mean ± SEM, *n* = 8–10 rats per group. Data were analyzed by Student’s paired *t*-test: different from T1: ^∗^*p* < 0.05; ^∗∗^*p* < 0.01.

In the matching-to-place version of the Morris water maze, on the test trial (T2) rats need to remember the location of the platform learned during the previous search trial (T1) that occurred 30 s or 2 min before. For an ITI of 30 s (**Figure 2A**), a two-way ANOVA on mean distance swum in T1 and T2 revealed no group effect (*F*_(4,76)_ = 1.59; *p* = 0.18) but a main effect of trial (*F*_(1,76)_ = 15.8; *p* < 0.001). Thus the distance swum for T2 was shorter than for T1 when data was collapsed across the group, suggesting that all groups remembered the location of the hidden platform learned at the previous trial. However, the two-way ANOVA also revealed a group × trial interaction (*F*_(4,76)_ = 2.9; *p* < 0.05). Therefore, when comparing T1 with T2, adult control rats and middle-aged rats supplemented with EPA/DHA exhibited distance swum in T2 significantly shorter than in T1 (paired *t*-test, *t* = 3.59, *p* = 0.005 and *t* = 4.27 *p* = 0.04, respectively), revealing better working memory performance in middle-aged rats supplemented with EPA/DHA. With a 2 min ITI (**Figure 2B**), there was no group effect (*F*_(4,76)_ = 0.9; *p* = 0.45) and the trial effect was just significant (*F*_(1,76)_ = 4.00; *p* = 0.05). The two-way ANOVA revealed a group × trial interaction (*F*_(4,76)_ = 2.6; *p* < 0.05) however, only the adult control rats swum a shorter distance during T2 than during T1, indicating that only adult control rats remembered the location of the platform (paired *t*-test, *t* = 2.97, *p* < 0.05). The EPA/DHA supplementation did not improve working memory in these conditions (paired *t*-test, *t* = 0.75, *p* = 0.47).

### Cued Learning

The distances swum to find the visible platform were not statistically significant different between the five different groups of rats tested in the cued version of the Morris water maze (*F*_(4,38)_ = 1.68; *p* = 0.17), indicating no difference in physical capabilities (**Figure 1D**).

### Nutritional Status of the Rats

#### Serum Retinol Concentration

**FIGURE 3:**
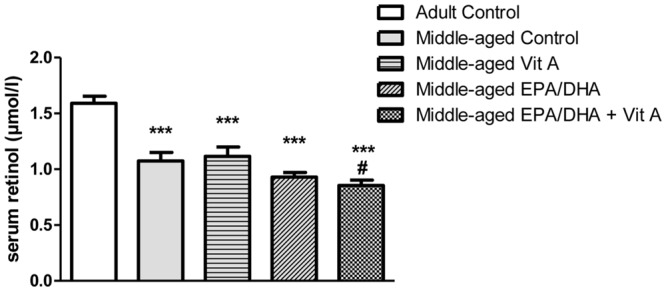
**Effect of age and diet (EPA/DHA, Vit A, or EPA/DHA + Vit A) on serum retinol concentrations.** Values are mean ± SEM, *n* = 8–10 rats per group. Data were analyzed by a one-way ANOVA followed by the Fischer PLSD *post hoc* test. Signs indicate values different from Adult control: ^∗∗∗^*p* < 0.001 and from middle-aged control: #*p* < 0.05.

In order to assess the vitamin A status of middle-aged rats and the impact of vitamin A supplementation, serum retinol concentrations were assessed (**Figure 3**). Our results showed a significant difference in retinol levels between groups (*F*_(4,38)_ = 20.76; *p* < 0.0001). Indeed, *post hoc* analysis revealed that middle-aged control group displayed lower serum retinol levels compared to the adult control group (-32%, *p* < 0.0001). Neither a vitamin A supplementation, nor an EPA/DHA supplementation, nor the combined supplementation were able to alleviate the age-related serum retinol decrease (-29%, *p* < 0.0001, -41%, *p* < 0.0001, and -46%, *p* < 0.0001, respectively). Interestingly, rats that received the EPA/DHA + Vit A diet displayed lower levels of retinol compared to middle-aged control rats (*p* = 0.024). The EPA/DHA group displayed an intermediate level of serum retinol between the vitamin A and the EPA/DHA + Vit A supplemented rats and differed only from the adult control rats.

#### Lipid Status in the Hippocampus

The results of the fatty acid composition of the hippocampal PC and PE are reported in **Tables 3** and **4** respectively. Aging was associated with a decrease in total n-3 LC-PUFAs only in hippocampal PC (-16%; *p* < 0.05) not in the PE, due to a decrease in DHA level (-17%; *p* < 0.05), leading to a slight elevation of the n-6 PUFA/n-3 PUFA ratio (+8%, *p* < 0.05) in middle-aged control group compared to the adult control group. Furthermore, our results showed a higher level of total monounsaturated fatty acids (MUFAs) in middle-aged control rats compared to adult control rats both in hippocampal PC and PE (+6 and +14%, respectively; *p* < 0.05). Dietary supplementation was associated with a similar increase in DHA levels in middle-aged EPA/DHA and in middle-aged EPA/DHA + Vit A supplemented rats both in hippocampal PC and PE (+21 and +26%, respectively for PC and +21 and +21% for PE respectively; all *p* < 0.05) compared to the middle-aged control group. DHA level in Vit A supplemented group was not different from that of adult control rats for both PC and PE. The EPA and its derivative n-3 docosapentaenoic acid (n-3 DPA) levels in hippocampal PC and PE were also strongly increased in middle-aged EPA/DHA and in middle-aged EPA/DHA + Vit A (EPA: +1200% for PC, *p* < 0.0001 and +730 and 560%, respectively for PE, *p* < 0.0001; n-3 DPA: +140% for PC, *p* < 0.0001 and +412% for PE, *p* < 0.0001) compared to the middle-aged control group. In contrast, AA and n-6 docosapentaenoic acid (n-6 DPA) contents in hippocampal PC and PE were reduced in middle-aged rats supplemented with EPA/DHA or EPA/DHA + Vit A (AA: -19% for PC and -16% for PE, *p* < 0.01; n-6 DPA: -48%, *p* < 0.05 and -39%, *p* < 0.0001, respectively for PC and -73% for PE *p* < 0.05) compared to the middle-aged control group. Thus, EPA/DHA supplementation induced a significant decrease in the n-6 PUFA/n-3 PUFA ratio for both PC and PE (-35 and -37%, respectively for PC and -40 and -38%, respectively for PE; *p* < 0.0001) compared to the middle-aged rats.

**Table 3:** Fatty acid composition (% of total fatty acids) of hippocampal phosphatidylcholine of rats fed the control diet or a supplemented diet (EPA/DHA, Vit A, or EPA/DHA + Vit A).

Fatty acids	Adult	Middle-aged			
	Control	Control	Vit A	EPA/DHA	EPA/DHA + Vit A
16:0	42.10 ± 0.73^a^	41.68 ± 0.63^a^	41.70 ± 0.53^a^	42.45 ± 0.32^a^	41.42 ± 0.82^a^
16:1(n-7)	0.38 ± 0.01**^a^**	0.43 ± 0.01**^b^**	0.44 ± 0.02**^bd^**	0.49 ± 0.02^cd^	0.50 ± 0.02^c^
18:0	12.78 ± 0.21^a^	12.97 ± 0.17^a^	12.83 ± 0.15^a^	12.74 ± 0.17^a^	12.98 ± 0.37^a^
18:1(n-9)	21.29 ± 0.34**^a^**	22.79 ± 0.40^bc^	22.43 ± 0.31**^ab^**	23.76 ± 0.38^c^	23.91 ± 0.41^c^
18:1(n-7)	5.98 ± 0.07**^a^**	5.97 ± 0.11**^a^**	5.89 ± 0.10**^a^**	5.41 ± 0.04^b^	5.47 ± 0.17^b^
18:2(n-6)	0.36 ± 0.02**^a^**	0.43 ± 0.03**^a^**	0.43 ± 0.02**^a^**	0.57 ± 0.03^b^	0.60 ± 0.04^b^
18:3(n-3)	ND	ND	ND	ND	ND
20:4(n-6) (AA)	9.06 ± 0.40**^a^**	8.06 ± 0.32**^b^**	8.21 ± 0.27**^ab^**	6.53 ± 0.29^c^	6.54 ± 0.46^c^
20:5(n-3) (EPA)	0.01 ± 0.01**^a^**	0.01 ± 0.01**^a^**	0.02 ± 0.01**^a^**	0.13 ± 0.01^b^	0.13 ± 0.02^b^
22:5(n-6) (DPA)	0.36 ± 0.04**^a^**	0.32 ± 0.06^ac^	0.41 ± 0.04**^a^**	0.17 ± 0.04^b^	0.19 ± 0.04^bc^
22:5(n-3) (DPA)	0.06 ± 0.01**^a^**	0.08 ± 0.01**^a^**	0.07 ± 0.01**^a^**	0.19 ± 0.01^b^	0.20 ± 0.02^b^
22:6(n-3) (DHA)	3.28 ± 0.20^a^	2.69 ± 0.16**^b^**	2.84 ± 0.13^ab^	3.26 ± 0.20^a^	3.39 ± 0.21^a^
Total SFAs	56.01 ± 0.55^a^	55.89 ± 0.53^a^	55.72 ± 0.49^a^	56.43 ± 0.37^a^	55.78 ± 0.36^a^
Total MUFAs	29.37 ± 0.42**^a^**	31.16 ± 0.49^b^	30.76 ± 0.42^ab^	31.47 ± 0.42^b^	31.76 ± 0.61^b^
Total PUFAs	14.43 ± 0.67**^a^**	12.78 ± 0.60^ab^	13.27 ± 0.46^ab^	11.90 ± 0.57^b^	12.20 ± 0.66^b^
Total n-6 PUFAs	10.91 ± 0.47**^a^**	9.83 ± 0.41**^b^**	10.11 ± 0.33**^ab^**	8.11 ± 0.35^c^	8.27 ± 0.43^c^
Total n-3 PUFAs	3.35 ± 0.20**^ac^**	2.79 ± 0.16**^b^**	2.93 ± 0.13**^ab^**	3.59 ± 0.22^c^	3.74 ± 0.24^c^
n-6/n-3 PUFAs	3.26 ± 0.07**^a^**	3.52 ± 0.07**^b^**	3.45 ± 0.08**^ab^**	2.26 ± 0.06^c^	2.21 ± 0.08^c^

*Values are mean ± SEM and were compared using one-way ANOVA and the Fischer PLSD *post hoc* test. Values not sharing a common superscript within a row are significantly different (*p* < 0.05). Underlined superscript indicates a statistically significant difference from the adult control group and bolded superscript indicates a statistically significant difference from the middle-aged EPA/DHA + Vit A group. ND, not detected; AA, arachidonic acid; DHA, docosahexaenoic acid; DPA, docosapentaenoic acid; EPA, eicosapentaenoic acid; SFA, saturated fatty acid; MUFA, monounsaturated fatty acid; PUFA, polyunsaturated fatty acid.*

**Table 4:** Fatty acid composition (% of total fatty acids) of hippocampal phosphatidylethanolamine of rats fed the control diet or a supplemented diet (EPA/DHA, Vit A, or EPA/DHA + Vit A).

Fatty acids	Adult	Middle-aged			
	Control	Control	Vit A	EPA/DHA	EPA/DHA + Vit A
16:00	5.83 ± 0.21^a^	5.98 ± 0.15^a^	5.84 ± 0.06^a^	6.2 ± 0.17^a^	6.07 ± 0.12^a^
16:1(n-7)	0.16 ± 0.01**^a^**	0.21 ± 0**^b^**	0.25 ± 0.03^bc^	0.3 ± 0.02^c^	0.29 ± 0.03^c^
18:00	18.44 ± 0.44^a^	18.14 ± 0.36^a^	17.87 ± 0.51^a^	17.51 ± 0.43^a^	17.99 ± 0.37^a^
18:1(n-9)	11.47 ± 0.35**^a^**	12.5 ± 0.23**^b^**	12.78 ± 0.44^bc^	13.91 ± 0.47^c^	13.63 ± 0.36^c^
18:1(n-7)	1.91 ± 0.08**^a^**	2.13 ± 0.06^b^	2.08 ± 0.09^ab^	2.13 ± 0.09^ab^	2.13 ± 0.08^b^
18:2(n-6)	0.14 ± 0.01**^a^**	0.17 ± 0.01**^ab^**	0.2 ± 0.02**^b^**	0.27 ± 0.01^c^	0.24 ± 0.01^c^
18:3(n-3)	ND	ND	ND	ND	ND
20:4(n-6) (AA)	13.98 ± 0.25**^a^**	13.38 ± 0.12**^a^**	13.6 ± 0.21**^a^**	11.2 ± 0.27^b^	11.24 ± 0.23^b^
20:5(n-3) (EPA)	0.02 ± 0.01**^a^**	0.03 ± 0.01**^a^**	0.04 ± 0.01**^a^**	0.25 ± 0.01**^b^**	0.2 ± 0.02^c^
22:5(n-6) (DPA)	1.01 ± 0.13**^a^**	0.99 ± 0.06**^a^**	0.98 ± 0.02**^a^**	0.26 ± 0.02^b^	0.26 ± 0.01^b^
22:5(n-3) (DPA)	0.13 0**^a^**	0.16 ± 0.02**^a^**	0.17 ± 0.01**^a^**	0.82 ± 0.03^b^	0.82 ± 0.02^b^
22:6(n-3) (DHA)	17.07 ± 0.58**^a^**	15.66 ± 0.41**^a^**	15.87 ± 0.29**^a^**	19.06 ± 0.71^b^	19.01 ± 0.46^b^
Total SFAs	39.57 ± 0.58^a^	38.38 ± 0.44^ab^	36.98 ± 0.59^b^	37.66 ± 0.46^b^	38.35 ± 0.47^ab^
Total MUFAs	21.56 ± 0.69**^a^**	24.64 ± 0.36^b^	24.62 ± 0.43^b^	25.73 ± 0.73^b^	24.89 ± 0.67^b^
Total PUFAs	38.49 ± 0.97^a^	36.67 ± 0.6^a^	37.6 ± 0.55^a^	36.19 ± 0.98^a^	36.36 ± 0.75^a^
Total n-6 PUFAs	21.06 ± 0.38**^a^**	20.55 ± 0.26**^a^**	21.11 ± 0.31**^a^**	15.8 ± 0.31^b^	16.04 ± 0.3^b^
Total n-3 PUFAs	17.22 ± 0.59**^a^**	15.85 ± 0.41**^a^**	16.11 ± 0.28**^a^**	20.14 ± 0.72^b^	20.04 ± 0.47^b^
n-6/n-3 PUFAs	1.22 ± 0.02**^a^**	1.3 ± 0.03**^a^**	1.31 ± 0.02**^a^**	0.78 ± 0.02^b^	0.8 ± 0.02^b^

*Values are mean ± SEM and were compared using one-way ANOVA and the Fischer PLSD *post hoc* test. Values not sharing a common superscript within a row are significantly different (*p* < 0.05). Underlined superscript indicates a statistically significant difference from the adult control group and bolded superscript indicates a statistically significant difference from the middle-aged EPA/DHA + Vit A group. ND, not detected; AA, arachidonic acid; DHA, docosahexaenoic acid; DPA, docosapentaenoic acid; EPA, eicosapentaenoic acid; SFA, saturated fatty acid; MUFA, monounsaturated fatty acid; PUFA, polyunsaturated fatty acid.*

### mRNA Expression in the Hippocampus

**FIGURE 4:**
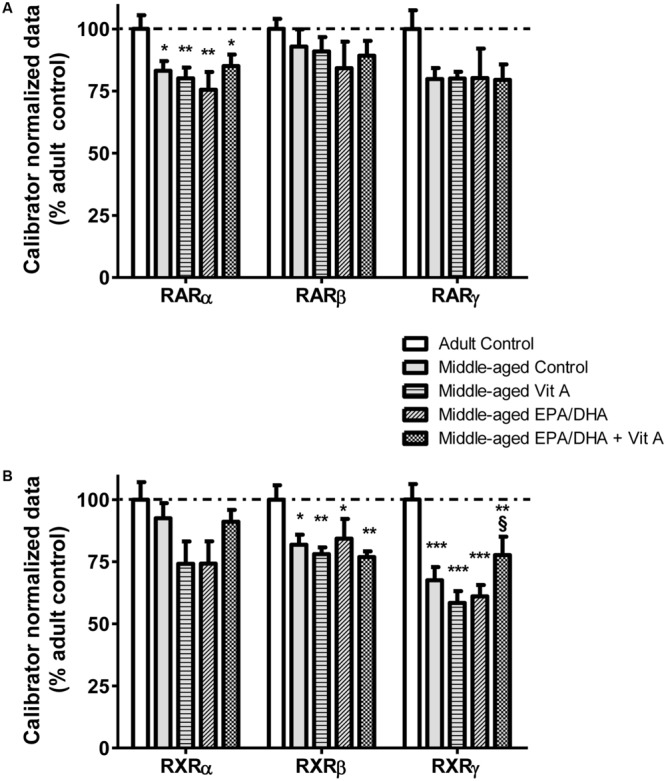
**Effect of age and diet (EPA/DHA, Vit A, or EPA/DHA + Vit A) on the mRNA expression of RARα, β, γ **(A)**, and RXRα, β, γ **(B)**.** The mRNA levels are expressed as the percentage of Adult control mRNA expression of target/reference ratio normalized by the calibrator. Values are mean ± SEM, *n* = 8–10 rats per group. Data were analyzed by one-way ANOVAs followed by the Fischer PLSD *post hoc* test. Signs indicate values different from Adult control: ^∗^*p* < 0.05; ^∗∗^*p* < 0.01; ^∗∗∗^*p* < 0.001 and from middle-aged Vit A and middle-aged EPA/DHA: §*p* < 0.05.

The mRNA expression of several PUFA and retinoid nuclear receptors was quantified by real-time PCR in the hippocampus. Results summarized in **Figure 4** show a significant group effect only for RARα, RXRβ and RXRγ mRNAs. Middle-aged rats exhibited a significant decrease in RARα (-17%; *F*_(4,37)_ = 3.29; *p* < 0.05) and RXRβ (-22%; *F*_(4,36)_ = 3.44; *p* < 0.05) mRNA expression in the hippocampus compared to the adult control rats. These mRNA levels were not modified with any dietary supplementations. RXRγ mRNA level was decreased in the middle-aged control group compared to the adult control group, the EPA/DHA group and the Vit A group (-32, -41, and -39% respectively; *F*_(4,35)_ = 7.98; *p* < 0.0001). However, the RXRγ mRNA level in the EPA/DHA + Vit A supplemented group was increased compared to the levels in Vit A and EPA/DHA supplemented groups (+33 and +27% respectively, *p* < 0.05).

**FIGURE 5:**
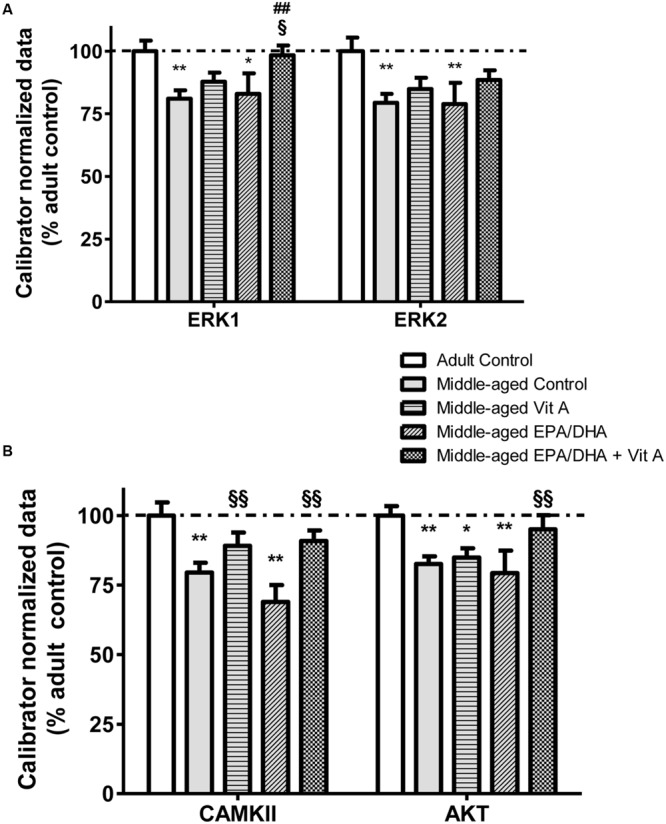
**Effect of age and diet (EPA/DHA, Vit A, or EPA/DHA + Vit A) on the mRNA expression of ERK1, ERK2 **(A)** and CAMKII and AKT **(B)**.** The mRNA levels are expressed as the percentage of Adult control mRNA expression of target/reference ratio normalized by the calibrator. Values are mean ± SEM, *n* = 8–10 rats per group. Data were analyzed by one-way ANOVAs followed by the Fischer PLSD *post hoc* test. Signs indicate values different from Adult control: ^∗^*p* < 0.05; ^∗∗^*p* < 0.01, from middle-aged control: ##*p* < 0.01 and from middle-aged EPA/DHA: §*p* < 0.05; §§*p* < 0.01.

The mRNA expression level coding for several proteins involved in both PUFA and RA extra-nuclear signaling pathways were also measured (**Figure 5**). An age-related hypo-expression of the mRNA levels of ERK1 (-19%; *F*_(4,37)_ = 3.228; *p* < 0.05), ERK2 (-21%; *F*_(4,37)_ = 2.668; *p* < 0.05), CAMKII (-21%; *F*_(4,37)_ = 6.602; *p* < 0.001) and AKT (-21%; *F*_(4,37)_ = 3.35; *p* < 0.05) was observed in the hippocampus of middle-aged control compared to adult control rats. Moreover, middle-aged rats supplemented with vitamin A (Vit A and EPA/DHA + Vit A) exhibited mRNA levels for ERK1, ERK2 and CAMKII not different from those of the adult control rats. Interestingly, for AKT, the hippocampal mRNA levels only in the middle-aged rats supplemented with EPA/DHA + Vit A were similar to those in adult control rats (95 vs. 100%; *p* = 0.47). Furthermore, only the mRNA level of ERK1 was significantly higher in the EPA/DHA + Vit A group compared to the middle-aged control group (+21%; *p* < 0.01).

### Protein Levels in the Hippocampus

**FIGURE 6:**
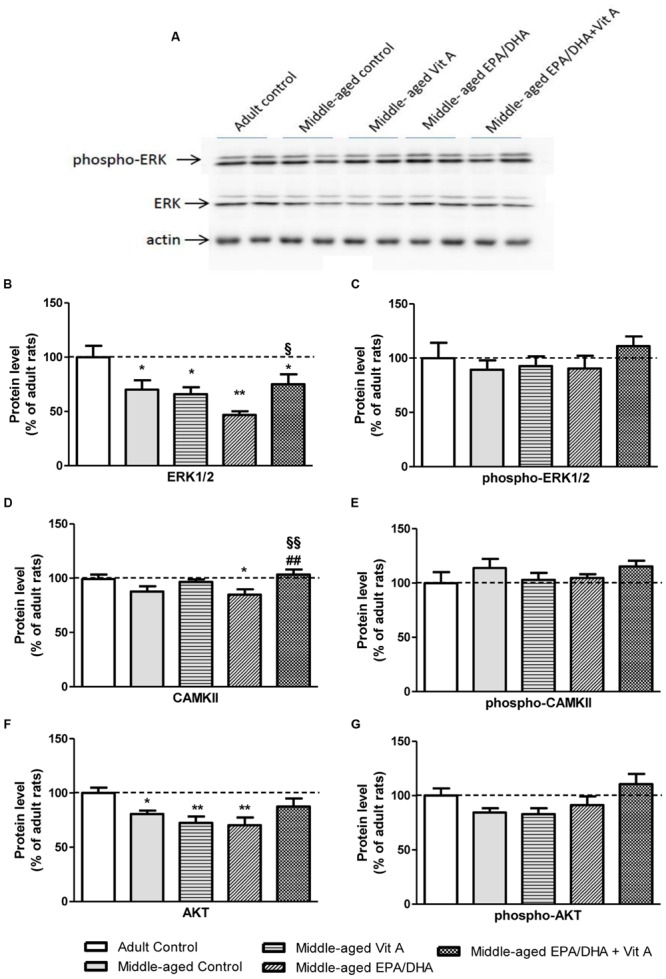
**Effect of age and diet (EPA/DHA, Vit A, or EPA/DHA + Vit A) on protein kinase expression in the hippocampus analyzed by Western blot.** The upper panel **(A)** illustrates representative immunoblots probed for phospho-Erk1/2, Erk1/2 and actin. Hippocampal Erk1/2 **(B)**, phospho-Erk1/2 **(C)**, CAMKII **(D)**, phospho-CAMKII **(E)**, AKT **(F)**, and phospho-AKT **(G)** protein levels were normalized against actin and expressed as a percentage of adult control expression of the same protein. Values are means ± SEM, *n* = 6–9 rats per group. Data were analyzed by one-way ANOVAs followed by the Fischer PLSD *post hoc* test. Signs indicate values different from Adult control: ^∗^*p* < 0.05; ^∗∗^*p* < 0.01; from middle-aged control: ##*p* < 0.01 and from middle-aged EPA/DHA: §*p* < 0.05; §§*p* < 0.01.

The results of the protein quantification by Western blot are reported in **Figure 6**. As for the mRNA expression, aging was associated with a significant reduced level of ERK1/2 (-30%, *F*_(4,32)_ = 4.639; *p* < 0.01) and AKT (-19%; *F*_(4,32)_ = 3.857; *p* < 0.05). Rats supplemented with EPA/DHA or vitamin A alone displayed similar protein levels of ERK1/2 and AKT compared to middle-aged control rats. However, the EPA/DHA + Vit A supplemented rats exhibited a protein level of AKT not significantly different from the one of adult control rats and an intermediated level of ERK1/2, indicating a additive effect of EPA/DHA and vitamin A supplementations. CAMKII protein level was not significantly different between adult and middle-aged control rats (*p* = 0.06). However, the EPA/DHA + Vit A supplemented rats exhibited a significantly higher level of CAMKII than the one of the middle-aged control rats (+18%; *p* < 0.01) and not different from adult control rats and Vit A supplemented rats. There was no statistical difference between groups in the expression levels of the phosphorylated forms of ERK1/2, CAMKII, and AKT in the hippocampus.

## Discussion

### Nutritional Status of the Rats

In the present study, middle-aged rats exhibited a significant decrease in serum retinol concentration compared to adult rats, as previously described in aged animals (van der Loo et al., 2004; Féart et al., 2005; Touyarot et al., 2013) and elderly people (Haller et al., 1996). This decrease could be explained by a loss, during aging, in the capacity to mobilize vitamin A from the liver and thereby to regulate serum retinol levels (Azais-Braesco et al., 1995; Borel et al., 1998). Thus, these changes in vitamin A bioavailability during aging could prevent the vitamin A-enriched diet to normalize the serum retinol level in middle-aged rats as previously shown in 13-month-old rats supplemented with 45 IU retinol/g of diet for 4 months (Touyarot et al., 2013).

The analyses of the fatty acid profile in the hippocampal PC revealed a significant decrease in DHA levels of middle-aged control rats compared to the adult control rats as previously described (Dyall et al., 2007; Letondor et al., 2014). However, contrary to the results of Dyall et al. (2007) we did not observe any age-related decrease in DHA levels in the hippocampal PE. This discrepancy could be explained by the very old age of the rats used by Dyall et al. (2007) (28 months in Dyall’s study vs. 18 months in the present study), leading to a drastic decrease in DHA levels in most phospholipid classes and in several brain structures. Aging was also associated with a significant increase in total MUFA levels both in hippocampal PC and PE as it was previously observed in the hippocampus and the cerebral cortex of 18-month-old rats (Favrelière et al., 2000). The EPA/DHA-enriched diet in middle-aged rats induced a strong increase in the n-3 LC-PUFA levels, including EPA, DHA and n-3 DPA, in both hippocampal PC and PE. This increase was counterbalanced with lower levels of n-6 LC-PUFAs leading to a decrease in the n-6 PUFA/n-3 PUFA ratio in the hippocampus. It is well known that fish oil diets containing n-3 LC-PUFAs reduce delta-6 and delta-5 desaturase activities leading to a decrease in n-6 LC-PUFAs (Christiansen et al., 1991). These data indicate that the age-related decrease in n-3 LC-PUFA levels in the hippocampal membranes can be reversed by the EPA/DHA-enriched diet as already reported in 21-month-old rats receiving a n-3 PUFA-enriched diet for 3 months (Favrelière et al., 2003).

The originality of the present study was to assess the effect of a 5-months vitamin A supplementation on brain fatty acid profile. Despite previous studies showing a regulation by vitamin A of hepatic desaturase expression involved in fatty acid metabolism (Zolfaghari and Ross, 2003), our results demonstrate that the vitamin A supplementation has no effect on fatty acid contents in the hippocampal PC or PE of middle-aged animals contrary to the EPA/DHA supplementation. This is in agreement with recent data showing that fat content in the diet has a stronger impact than vitamin A on fatty acid concentrations in the liver and in the plasma (Weiss et al., 2014).

### Cognitive Status and Molecular Mechanisms

#### Effect of Age

According to numerous data obtained in aged animals (Lister and Barnes, 2009), we demonstrated a clear age-related impairment in hippocampus-dependent spatial reference memory and spatial working memory in 18-month-old rats. These age-related memory impairments were associated with a hippocampal hypoexpression of RARα, RXRβ, and RXRγ mRNAs in middle-aged control animals. These results are consistent with previous data obtained in 23-month-old mice, showing a reduced level of RXRβ/γ mRNA expression in the hippocampus and in the whole brain (Enderlin et al., 1997a), that was associated with a relational memory impairment (Etchamendy et al., 2001). It is well known that the mRNA expression of some RXR and RAR isotypes is regulated by RA in the brain (Enderlin et al., 1997b; Féart et al., 2005) and that RA administration reverses an age-related spatial memory deficit (Etchamendy et al., 2001). In the rodent hippocampus, RA can be synthetized, from circulating vitamin A, in the meninges, by the retinaldehyde dehydrogenase 2 (Goodman et al., 2012). Therefore an age-related decrease in serum retinol concentration could lead to a decrease in RA synthesize in the brain, leading to a decrease in RAR and RXR expression in the hippocampus as previously shown in vitamin A deficiency models (Husson et al., 2004; Navigatore-Fonzo et al., 2013).

In addition, disruption of the RXR signaling pathway could be due to a potential decrease in intracellular DHA bioavailability. Indeed, besides the decrease in the DHA level in hippocampal membranes of aged rats, it has been reported a decrease in the independent phospholipase A2 (iPLA2) mRNA expression in the hippocampus of 24-month-old rats compared to 4-month-old rats (Aid and Bosetti, 2007). Knowing that iPLA2 is involved in the release of DHA from membrane phospholipids, this could induce a decrease in the content of DHA in the cell which notably can activate the RXR (de Urquiza et al., 2000). Several studies support the hypothesis that RXRγ plays a key role in learning and memory processes. Indeed, using a gene microarray approach, Blalock et al. (2003) have shown an age-related down-regulation of RXRγ mRNA level in hippocampal CA1 region positively correlated with impaired hippocampus-dependent memory performance. Furthermore, RXRγ knock-out mice display working memory impairments and a pharmacological administration of unesterified DHA improves working memory performance of 5-month-old mice *via* the RXRγ activation (Wietrzych et al., 2005; Wietrzych-Schindler et al., 2011). At the mechanistic level, an *in vitro* study showed that a RXR activation induced by a DHA treatment was associated with a stimulation of neuritogenesis in mice neuroblastoma cells (Calderon and Kim, 2007), demonstrating that RXRs are involved in the modulation of synaptic plasticity. These data support the hypothesis that the age-related memory impairments observed in the present study may be closely linked to the disruption of retinoid and n-3 PUFA signaling pathways, involving notably the RXRγ.

Moreover, the present results showed a decrease in hippocampal CAMKII mRNA expression in middle-aged rats. CAMKII is an ubiquitous kinase in the brain, involved in the regulation of the strengthening synaptic transmission and therefore learning and memory processes (Giese and Mizuno, 2013). Since CAMKII mRNA expression is regulated by RA (Chen and Kelly, 1996), this last result supports the hypothesis of a retinoid signaling pathway disruption in the aged brain that may be responsible, in part, of the memory impairments by altering kinase signaling pathways. Furthermore, the present results demonstrated a decrease in mRNA and protein levels of AKT and ERK1/2 which can be activated by the retinoids and the DHA as a result of a non-genomic effect of these nutrients (Eady et al., 2012; Al Tanoury et al., 2013; Jiang et al., 2013). An age-related disruption of AKT and ERK1/2 signaling has already been observed in rodents. Indeed, Simonyi et al. (2003) reported a decrease in ERK2 mRNA expression in the hippocampus of 12-month-old rats compared to 3-month-old rats. Moreover a reduced AKT signaling pathway activation was observed in the hippocampus of 28-month-old rats compared to 4-month-old rats (Jackson et al., 2009) and in the hippocampus of senescence-accelerated mice (Nie et al., 2009; Armbrecht et al., 2014). These two signaling pathways play major roles in brain functioning since AKT signaling pathway is involved in neuronal survival (Kaplan and Miller, 2000) and ERK1/2 signaling pathway plays a role in hippocampus-dependent learning and memory (Xia and Storm, 2012). Thus the concomitant decrease in ERK1/2 and AKT expression in the hippocampus of middle-aged rats could be responsible, in part, of the age-related spatial memory impairments. Interestingly, the phosphorylation levels of these kinases were not modified in middle-aged rats. It should be noted that kinase activation occurs rapidly and transiently following the induction of synaptic potentiation consecutive to the learning process and constitute a short step before protein synthesis necessary to long term memory formation (Davis and Laroche, 2006). The present result could be due to our experimental settings, notably a 24-h delay between the end of the behavioral procedure and the euthanasia.

#### Effect of Nutritional Supplementations

Middle-aged rats supplemented with vitamin A displayed memory performance significantly above the chance level, confirming the beneficial effect of a long-term vitamin A supplementation on the aged-related spatial reference memory deficits in rats (Touyarot et al., 2013) or relational memory deficits in mice (Mingaud et al., 2008). This beneficial effect on memory occurred in spite of the decrease in serum retinol concentration measured in middle-aged rats supplemented with vitamin A. This result supports the hypothesis that the newly-absorbed retinol could be used directly by the target tissues such as the brain in order to cover RA needs by an *in situ* synthesis, as it was previously suggested (Ross et al., 2009; Goodman et al., 2012; Touyarot et al., 2013).

The main result of the present study highlights for the first time a beneficial additive effect of EPA/DHA and vitamin A supplementation on the reference memory since only the adult and the middle-aged EPA/DHA + Vit A supplemented rats exhibited performance above the chance level in the probe test over 60 s. In order to avoid an extinction effect due to the absence of the platform (Blokland et al., 2004), we also performed analyses only over the first third period of the probe test. In this case, we observed a stronger effect since only the middle-aged EPA/DHA + Vit A supplemented rats exhibited performance closer to those of the adult control rats. On the contrary, the memory performance of either vitamin A or EPA/DHA supplemented rats were still significantly lower than those of adult control rats. Moreover, this combined nutritional supplementation partially alleviated the age-related decrease only in RXRγ mRNA expression in the hippocampus of middle-aged rats. Contrary to our results, Dyall et al. (2010) have shown that a fish oil supplementation for 12 weeks in 25–26 month-old rats reverses the age-related decrease in RARα, RXRα, and RXRβ protein levels in the CA1 and the dentate gyrus of the hippocampus, using an immunohistochemical approach. This discrepancy in fish oil effect on nuclear receptor expression could be explained by the dose or the duration of the fish oil supplementation but also by the age of the rats that are different between the two studies. Moreover, in the present study we measured RAR and RXR expression at the mRNA level in the whole hippocampus contrary to Dyall et al. (2010) who quantified protein levels in discrete hippocampal regions. Therefore, a dilution effect could explain this discrepancy and the use of an *in situ* hybridization approach would permit to compare the two studies. However, this result is consistent with data supporting the major role played by RXRγ in memory processes as discussed above. Moreover, EPA/DHA + Vit A supplemented middle-aged rats exhibited a maintenance of AKT mRNA and protein expression in the hippocampus. AKT signaling pathway is involved in neuronal survival (Kaplan and Miller, 2000) and the beneficial role of DHA on neuronal survival mediated by the AKT signaling pathway has already been reported (Akbar et al., 2005). Vitamin A can also induce the embryonic stem cell renewal *via* an activation of the AKT signaling pathway (Chen and Khillan, 2010). However, to our knowledge, the present study is the first one that demonstrates a combined effect of n-3 LC-PUFAs and vitamin A supplemented diet on AKT in the hippocampus. Furthermore, the two vitamin A supplemented groups exhibited CAMKII and ERK1/2 mRNA and protein levels similar to those measured in the adult control group. According to the involvement of these kinases in learning and memory, it could be hypothesized that their maintenance during aging could participate to the beneficial effect of vitamin A on the reference memory in middle-aged rats (Giese and Mizuno, 2013). As previously shown for CAMKII, the transcriptional regulation of these kinases could be mediated by retinoids (Chen and Kelly, 1996). Since this is the first study investigating the effects of a combined EPA/DHA and vitamin A supplementation, no experimental data could yet explain this additive effect. However, a study performed in mouse neuroblastoma cells showed that the iPLA2 activity, involved in the release of DHA from membrane phospholipids, in nuclear membrane was induced by a RA treatment (Farooqui et al., 2004). According to this result, it can be hypothesized that vitamin A, the precursor of RA, could potentiate the effects of the EPA/DHA supplementation by increasing intracellular DHA bioavailability leading to a maintenance of RXRγ and kinase signaling pathways necessary for optimal memory processes.

The single EPA/DHA supplementation was not able to prevent the age-related reference memory deficits, as it has already been reported in studies performed with the same diet duration. Indeed, aged rats receiving 140 mg/kg body weight EPA and 109 mg/kg body weight DHA displayed a spatial reference memory enhancement in a eight-arm radial maze (Hashimoto et al., 2015) and aged mice receiving 0.9–23.7 g of DHA/100 g of fatty acids showed an enhancement of maze-learning ability (Lim and Suzuki, 2000). Nevertheless, the present results point out a beneficial effect of n-3 LC-PUFA supplementation on working memory performance assessed over a short retention delay (ITI of 30 s). These results are in accordance with previous data obtained in our laboratory showing that an EPA/DHA supplementation for 4 months in 13-month-old rats prevented the age-related working memory deficits with an ITI of 30 s (Alfos et al., unpublished data).

Interestingly, we demonstrated that the beneficial effect mediated by n-3 LC-PUFA supplementation remained limited since no memory improvement was observed in supplemented middle-aged rats with an ITI of 2 min. There was no effect of the EPA/DHA supplemented diet on the mRNA and the protein levels measured in the hippocampus contrary to the effects observed with the diet enriched with vitamin A, suggesting that the EPA/DHA effect on working memory seems to be mediated by another molecular signaling pathway.

## Conclusion

Taken together, this study demonstrated impairments in reference memory and working memory, associated with n-3 fatty acid and vitamin A metabolism alterations and a decrease in RXRβ and RXRγ mRNAs and CAMKII, AKT, ERK1/2 expression in the hippocampus of middle-aged rats. Our results highlight for the first time a preventive additive effect of an EPA/DHA-and vitamin A-enriched diet on the age-related decline in reference memory performance. This beneficial effect on memory could be in part mediated both by RXRγ and kinase signaling pathways that were maintained in the hippocampus of middle-aged supplemented rats. Our findings provide new targets within the framework of preventive nutrition to delay brain aging and demonstrate that combinations of dietary nutrients need to be more fully evaluated to determine optimal strategies as recently suggested by Casali et al. (2015) that used a combined therapy with DHA and bexarotene, a RXR agonist, in a mouse model of Alzheimer’s disease.

## Author Contributions

AL, SA, and BB analyzed the data and wrote the manuscript. AL, SA, ER performed the experiments. BB, CV, SL, VP, and SA designed the study and supervised the work.

## Conflict of Interest Statement

The authors declare that the research was conducted in the absence of any commercial or financial relationships that could be construed as a potential conflict of interest.

## Abbreviations

AA: arachidonic acid
AKT: protein kinase B
CAMKII: calcium/calmodulin-dependent kinase II
DHA: docosahexaenoic acid
EPA: eicosapentaenoic acid
ERK: extracellular signal-regulated kinase
MAPK: mitogen-activated protein kinase
n-3 LC-PUFA: n-3 long-chain polyunsaturated fatty acid
PC: phosphatidylcholine
PE: phosphatidylethanolamine
PPAR: peroxisome proliferator-activated receptor
RA: retinoic acid
RAR: retinoic acid receptor
RXR: retinoid X receptor

## References

[B1] Aid S. Bosetti F. (2007). Gene expression of cyclooxygenase-1 and Ca(2+)-independent phospholipase A(2) is altered in rat hippocampus during normal aging. *Brain Res. Bull.* 73 108–113. 10.1016/j.brainresbull.2007.02.015 17499644 PMC1945113

[B2] Akbar M. Calderon F. Wen Z. Kim H. Y. (2005). Docosahexaenoic acid: a positive modulator of Akt signaling in neuronal survival. *Proc. Natl. Acad. Sci. U.S.A.* 102 10858–10863. 10.1073/pnas.0502903102 16040805 PMC1182431

[B3] Al Tanoury Z. Piskunov A. Rochette-Egly C. (2013). Vitamin A and retinoid signaling: genomic and nongenomic effects. *J. Lipid Res.* 54 1761–1775. 10.1194/jlr.R030833 23440512 PMC3679380

[B4] Alfos S. (2014). “Fish oil supplementation prevents age-related memory decline: involvement of nuclear hormone receptors,” in *Omega 3 Fatty Acids in Brain and Neurologic Health* 1st Edn eds Watson R. R. De Meester F. (San Diego, CA: Elsevier) 147–161.

[B5] Armbrecht H. J. Siddiqui A. M. Green M. Farr S. A. Kumar V. B. Banks W. A. (2014). SAMP8 mice have altered hippocampal gene expression in long term potentiation, phosphatidylinositol signaling, and endocytosis pathways. *Neurobiol. Aging* 35 159–168. 10.1016/j.neurobiolaging.2013.07.018 23969180 PMC3839577

[B6] Azais-Braesco V. Dodeman I. Delpal S. Alexandre-Gouabau M. C. Partier A. Borel P. (1995). Vitamin A contained in the lipid droplets of rat liver stellate cells is substrate for acid retinyl ester hydrolase. *Biochim. Biophys. Acta* 1259 271–276. 10.1016/0005-2760(95)00173-5 8541334

[B7] Blalock E. M. Chen K. C. Sharrow K. Herman J. P. Porter N. M. Foster T. C. (2003). Gene microarrays in hippocampal aging: statistical profiling identifies novel processes correlated with cognitive impairment. *J. Neurosci.* 23 3807–3819. 12736351 10.1523/JNEUROSCI.23-09-03807.2003 PMC6742177

[B8] Blokland A. Geraerts E. Been M. (2004). A detailed analysis of rats’ spatial memory in a probe trial of a Morris task. *Behav. Brain Res.* 154 71–75. 10.1016/j.bbr.2004.01.022 15302112

[B9] Bonhomme D. Pallet V. Dominguez G. Servant L. Henkous N. Lafenetre P. (2014). Retinoic acid modulates intrahippocampal levels of corticosterone in middle-aged mice: consequences on hippocampal plasticity and contextual memory. *Front. Aging Neurosci.* 6:6 10.3389/fnagi.2014.00006 PMC3917121 24570662

[B10] Bonnet E. Touyarot K. Alfos S. Pallet V. Higueret P. Abrous D. N. (2008). Retinoic acid restores adult hippocampal neurogenesis and reverses spatial memory deficit in vitamin A deprived rats. *PLoS ONE* 3:e3487 10.1371/journal.pone.0003487 PMC2567033 18941534

[B11] Borel P. Mekki N. Boirie Y. Partier A. Alexandre-Gouabau M. C. Grolier P. (1998). Comparison of the postprandial plasma vitamin A response in young and older adults. *J. Gerontol. A Biol. Sci. Med. Sci.* 53 B133–B140. 10.1093/gerona/53A.2.B133 9520909

[B12] Boucheron C. Alfos S. Enderlin V. Husson M. Pallet V. Jaffard R. (2006). Age-related effects of ethanol consumption on triiodothyronine and retinoic acid nuclear receptors, neurogranin and neuromodulin expression levels in mouse brain. *Neurobiol. Aging* 27 1326–1334. 10.1016/j.neurobiolaging.2005.07.008 16115698

[B13] Buaud B. Esterle L. Vaysse C. Alfos S. Combe N. Higueret P. (2010). A high-fat diet induces lower expression of retinoid receptors and their target genes GAP-43/neuromodulin and RC3/neurogranin in the rat brain. *Br. J. Nutr.* 103 1720–1729. 10.1017/S0007114509993886 20102671

[B14] Calderon F. Kim H. Y. (2007). Role of RXR in neurite outgrowth induced by docosahexaenoic acid. *Prostaglandins Leukot. Essent. Fatty Acids* 77 227–232. 10.1016/j.plefa.2007.10.026 18036800 PMC2174793

[B15] Casali B. T. Corona A. W. Mariani M. M. Karlo J. C. Ghosal K. Landreth G. E. (2015). Omega-3 fatty acids augment the actions of nuclear receptor agonists in a mouse model of alzheimer’s disease. *J. Neurosci.* 35 9173–9181. 10.1523/JNEUROSCI.1000-15.2015 26085639 PMC4469742

[B16] Chen J. Kelly P. T. (1996). Retinoic acid stimulates alpha-CAMKII gene expression in PC12 cells at a distinct transcription initiation site. *J. Neurosci.* 16 5704–5714. 8795626 10.1523/JNEUROSCI.16-18-05704.1996 PMC6578957

[B17] Chen L. Khillan J. S. (2010). A novel signaling by vitamin A/retinol promotes self renewal of mouse embryonic stem cells by activating PI3K/Akt signaling pathway via insulin-like growth factor-1 receptor. *Stem Cells* 28 57–63. 10.1002/stem.251 19890980

[B18] Christiansen E. N. Lund J. S. Rortveit T. Rustan A. C. (1991). Effect of dietary n-3 and n-6 fatty acids on fatty acid desaturation in rat liver. *Biochim. Biophys. Acta* 1082 57–62. 10.1016/0005-2760(91)90299-W 2009302

[B19] Davis S. Laroche S. (2006). Mitogen-activated protein kinase/extracellular regulated kinase signalling and memory stabilization: a review. *Genes Brain Behav.* 5(Suppl. 2) 61–72. 10.1111/j.1601-183X.2006.00230.x 16681801

[B20] de Urquiza A. M. Liu S. Sjoberg M. Zetterstrom R. H. Griffiths W. Sjovall J. (2000). Docosahexaenoic acid, a ligand for the retinoid X receptor in mouse brain. *Science* 290 2140–2144. 10.1126/science.290.5499.2140 11118147

[B21] Dyall S. C. Michael G. J. Michael-Titus A. T. (2010). Omega-3 fatty acids reverse age-related decreases in nuclear receptors and increase neurogenesis in old rats. *J. Neurosci. Res.* 88 2091–2102. 10.1002/jnr.22390 20336774

[B22] Dyall S. C. Michael G. J. Whelpton R. Scott A. G. Michael-Titus A. T. (2007). Dietary enrichment with omega-3 polyunsaturated fatty acids reverses age-related decreases in the GluR2 and NR2B glutamate receptor subunits in rat forebrain. *Neurobiol. Aging* 28 424–439. 10.1016/j.neurobiolaging.2006.01.002 16500747

[B23] Eady T. N. Belayev L. Khoutorova L. Atkins K. D. Zhang C. Bazan N. G. (2012). Docosahexaenoic acid signaling modulates cell survival in experimental ischemic stroke penumbra and initiates long-term repair in young and aged rats. *PLoS ONE* 7:e46151 10.1371/journal.pone.0046151 PMC3484151 23118851

[B24] Enderlin V. Alfos S. Pallet V. Garcin H. Azais-Braesco V. Jaffard R. (1997a). Aging decreases the abundance of retinoic acid (RAR) and triiodothyronine (TR) nuclear receptor mRNA in rat brain: effect of the administration of retinoids. *FEBS Lett.* 412 629–632. 10.1016/S0014-5793(97)00845-4 9276480

[B25] Enderlin V. Pallet V. Alfos S. Dargelos E. Jaffard R. Garcin H. (1997b). Age-related decreases in mRNA for brain nuclear receptors and target genes are reversed by retinoic acid treatment. *Neurosci. Lett.* 229 125–129. 10.1016/S0304-3940(97)00424-2 9223607

[B26] Etchamendy N. Enderlin V. Marighetto A. Vouimba R. M. Pallet V. Jaffard R. (2001). Alleviation of a selective age-related relational memory deficit in mice by pharmacologically induced normalization of brain retinoid signaling. *J. Neurosci.* 21 6423–6429. 11487666 10.1523/JNEUROSCI.21-16-06423.2001 PMC6763177

[B27] Evans R. M. Mangelsdorf D. J. (2014). Nuclear receptors, RXR, and the big bang. *Cell* 157 255–266. 10.1016/j.cell.2014.03.012 24679540 PMC4029515

[B28] Farooqui A. A. Antony P. Ong W. Y. Horrocks L. A. Freysz L. (2004). Retinoic acid-mediated phospholipase A(2) signaling in the nucleus. *Brain Res. Rev.* 45 179–195. 10.1016/j.brainresv.2004.03.002 15210303

[B29] Favrelière S. Perault M. C. Huguet F. De Javel D. Bertrand N. Piriou A. (2003). DHA-enriched phospholipid diets modulate age-related alterations in rat hippocampus. *Neurobiol. Aging* 24 233–243. 10.1016/S0197-4580(02)00064-7 12498957

[B30] Favrelière S. Stadelmann-Ingrand S. Huguet F. De Javel D. Piriou A. Tallineau C. (2000). Age-related changes in ethanolamine glycerophospholipid fatty acid levels in rat frontal cortex and hippocampus. *Neurobiol. Aging* 21 653–660. 10.1016/S0197-4580(00)00170-6 11016534

[B31] Féart C. Vallortigara J. Higueret D. Gatta B. Tabarin A. Enderlin V. (2005). Decreased expression of retinoid nuclear receptor (RARα and RARγ) mRNA determined by real-time quantitative RT-PCR in peripheral blood mononuclear cells of hypothyroid patients. *J. Mol. Endocrinol.* 34 849–858. 10.1677/jme.1.01662 15956352

[B32] Giese K. P. Mizuno K. (2013). The roles of protein kinases in learning and memory. *Learn. Mem.* 20 540–552. 10.1101/lm.028449.112 24042850

[B33] Goodman T. Crandall J. E. Nanescu S. E. Quadro L. Shearer K. Ross A. (2012). Patterning of retinoic acid signaling and cell proliferation in the hippocampus. *Hippocampus* 22 2171–2183. 10.1002/hipo.22037 22689466 PMC3505796

[B34] Haller J. Weggemans R. M. Lammi-Keefe C. J. Ferry M. (1996). Changes in the vitamin status of elderly Europeans: plasma vitamins A, E, B-6, B-12, folic acid and carotenoids. SENECA Investigators. *Eur. J. Clin. Nutr.* 50(Suppl. 2) S32–S46. 8841783

[B35] Hashimoto M. Katakura M. Tanabe Y. Al Mamun A. Inoue T. Hossain S. (2015). n-3 fatty acids effectively improve the reference memory-related learning ability associated with increased brain docosahexaenoic acid-derived docosanoids in aged rats. *Biochim. Biophys. Acta* 1851 203–209. 10.1016/j.bbalip.2014.10.009 25450447

[B36] Husson M. Enderlin V. Alfos S. Boucheron C. Pallet V. Higueret P. (2004). Expression of neurogranin and neuromodulin is affected in the striatum of vitamin A-deprived rats. *Brain Res. Mol. Brain Res.* 123 7–17. 10.1016/j.molbrainres.2003.12.012 15046861

[B37] Jackson T. C. Rani A. Kumar A. Foster T. C. (2009). Regional hippocampal differences in AKT survival signaling across the lifespan: implications for CA1 vulnerability with aging. *Cell Death Differ.* 16 439–448. 10.1038/cdd.2008.171 19039330 PMC2680608

[B38] Jiang L. H. Yan S. Wang J. Liang Q. Y. (2013). Oral administration of docosahexaenoic acid activates the GDNF-MAPK-CERB pathway in hippocampus of natural aged rat. *Pharm. Biol.* 51 1188–1195. 10.3109/13880209.2013.784341 23767459

[B39] Joffre C. Nadjar A. Lebbadi M. Calon F. Laye S. (2014). n-3 LCPUFA improves cognition: the young, the old and the sick. *Prostaglandins Leukot. Essent. Fatty Acids* 91 1–20. 10.1016/j.plefa.2014.05.001 24908517

[B40] Kaplan D. R. Miller F. D. (2000). Neurotrophin signal transduction in the nervous system. *Curr. Opin. Neurobiol.* 10 381–391. 10.1016/S0959-4388(00)00092-1 10851172

[B41] Labrousse V. F. Nadjar A. Joffre C. Costes L. Aubert A. Gregoire S. (2012). Short-term long chain omega3 diet protects from neuroinflammatory processes and memory impairment in aged mice. *PLoS ONE* 7:e36861 10.1371/journal.pone.0036861 PMC3360741 22662127

[B42] Lane M. A. Bailey S. J. (2005). Role of retinoid signalling in the adult brain. *Prog. Neurobiol.* 75 275–293. 10.1016/j.pneurobio.2005.03.002 15882777

[B43] Leclercq M. Bourgeay-Causse M. (1981). A simple, reliable fast method: simultaneous proportioning of retinol and serum tocopherol by high performance liquid chromatography. *Rev. Inst. Pasteur Lyon* 14 475–496.

[B44] Lengqvist J. Mata De Urquiza A. Bergman A. C. Willson T. M. Sjovall J. Perlmann T. (2004). Polyunsaturated fatty acids including docosahexaenoic and arachidonic acid bind to the retinoid X receptor alpha ligand-binding domain. *Mol. Cell. Proteomics* 3 692–703. 10.1074/mcp.M400003-MCP200 15073272

[B45] Letondor A. Buaud B. Vaysse C. Fonseca L. Herrouin C. Servat B. (2014). Erythrocyte DHA level as a biomarker of DHA status in specific brain regions of n-3 long-chain PUFA-supplemented aged rats. *Br. J. Nutr.* 112 1805–1818. 10.1017/S0007114514002529 25331622

[B46] Lim S.-Y. Suzuki H. (2000). Intakes of dietary docosahexaenoic acid ethyl ester and egg phosphatidylcholine improve maze-learning ability in young and old mice. *J. Nutr.* 130 1629–1632. 10827221 10.1093/jn/130.6.1629

[B47] Lister J. P. Barnes C. A. (2009). Neurobiological changes in the hippocampus during normative aging. *Arch. Neurol.* 66 829–833. 10.1001/archneurol.2009.125 19597084

[B48] Ma H. Li B. Tsien R. W. (2015). Distinct roles of multiple isoforms of CaMKII in signaling to the nucleus. *Biochim. Biophys. Acta* 1853 1953–1957. 10.1016/j.bbamcr.2015.02.008 25700840 PMC4522395

[B49] Masia S. Alvarez S. de Lera A. R. Barettino D. (2007). Rapid, nongenomic actions of retinoic acid on phosphatidylinositol-3-kinase signaling pathway mediated by the retinoic acid receptor. *Mol. Endocrinol.* 21 2391–2402. 10.1210/me.2007-0062 17595318

[B50] Mingaud F. Mormede C. Etchamendy N. Mons N. Niedergang B. Wietrzych M. (2008). Retinoid hyposignaling contributes to aging-related decline in hippocampal function in short-term/working memory organization and long-term declarative memory encoding in mice. *J. Neurosci.* 28 279–291. 10.1523/JNEUROSCI.4065-07.2008 18171945 PMC6671152

[B51] Navigatore-Fonzo L. S. Golini R. L. Ponce I. T. Delgado S. M. Plateo-Pignatari M. G. Gimenez M. S. (2013). Retinoic acid receptors move in time with the clock in the hippocampus. Effect of a vitamin-A-deficient diet. *J. Nutr. Biochem.* 24 859–867. 10.1016/j.jnutbio.2012.05.006 22902328 PMC3504648

[B52] Nie K. Yu J. C. Fu Y. Cheng H. Y. Chen F. Y. Qu Y. (2009). Age-related decrease in constructive activation of Akt/PKB in SAMP10 hippocampus. *Biochem. Biophys. Res. Commun.* 378 103–107. 10.1016/j.bbrc.2008.11.010 19013131

[B53] Parletta N. Milte C. M. Meyer B. J. (2013). Nutritional modulation of cognitive function and mental health. *J. Nutr. Biochem.* 24 725–743. 10.1016/j.jnutbio.2013.01.002 23517914

[B54] Rao J. S. Ertley R. N. Lee H. J. DeMar J. C. Jr. Arnold J. T. Rapoport S. I. (2007). n-3 polyunsaturated fatty acid deprivation in rats decreases frontal cortex BDNF via a p38 MAPK-dependent mechanism. *Mol. Psychiatry* 12 36–46. 10.1038/sj.mp.4001888 16983391

[B55] Ross A. C. Russell R. M. Miller S. A. Munro I. C. Rodricks J. V. Yetley E. A. (2009). Application of a key events dose-response analysis to nutrients: a case study with vitamin A (retinol). *Crit. Rev. Food Sci. Nutr.* 49 708–717. 10.1080/10408390903098749 19690996 PMC2840874

[B56] Schug T. T. Berry D. C. Shaw N. S. Travis S. N. Noy N. (2007). Opposing effects of retinoic acid on cell growth result from alternate activation of two different nuclear receptors. *Cell* 129 723–733. 10.1016/j.cell.2007.02.050 17512406 PMC1948722

[B57] Shaw N. Elholm M. Noy N. (2003). Retinoic acid is a high affinity selective ligand for the peroxisome proliferator-activated receptor beta/delta. *J. Biol. Chem.* 278 41589–41592. 10.1074/jbc.C300368200 12963727

[B58] Simoes A. E. Pereira D. M. Amaral J. D. Nunes A. F. Gomes S. E. Rodrigues P. M. (2013). Efficient recovery of proteins from multiple source samples after TRIzol((R)) or TRIzol((R))LS RNA extraction and long-term storage. *BMC Genomics* 14:181 10.1186/1471-2164-14-181 PMC3620933 23496794

[B59] Simonyi A. Murch K. Sun G. Y. (2003). Extracellular signal-regulated kinase 2 mRNA expression in the rat brain during aging. *Neurochem. Res.* 28 1375–1378. 10.1023/A:1024948532633 12938860

[B60] Su H. M. (2010). Mechanisms of n-3 fatty acid-mediated development and maintenance of learning memory performance. *J. Nutr. Biochem.* 21 364–373. 10.1016/j.jnutbio.2009.11.003 20233652

[B61] Touyarot K. Bonhomme D. Roux P. Alfos S. Lafenetre P. Richard E. (2013). A mid-life vitamin A supplementation prevents age-related spatial memory deficits and hippocampal neurogenesis alterations through CRABP-I. *PLoS ONE* 8:e72101 10.1371/journal.pone.0072101 PMC3747058 23977218

[B62] van der Loo B. Labugger R. Aebischer C. P. Bachschmid M. Spitzer V. Kilo J. (2004). Age-related changes of vitamin A status. *J. Cardiovasc. Pharmacol.* 43 26–30. 10.1097/00005344-200401000-00005 14668564

[B63] van Neerven S. Kampmann E. Mey J. (2008). RAR/RXR and PPAR/RXR signaling in neurological and psychiatric diseases. *Prog. Neurobiol.* 85 433–451. 10.1016/j.pneurobio.2008.04.006 18554773

[B64] Wainwright P. E. Xing H. C. Ward G. R. Huang Y. S. Bobik E. Auestad N. (1999). Water maze performance is unaffected in artificially reared rats fed diets supplemented with arachidonic acid and docosahexaenoic acid. *J. Nutr.* 129 1079–1089. 10222403 10.1093/jn/129.5.1079

[B65] Weiss K. Mihaly J. Liebisch G. Marosvolgyi T. Garcia A. L. Schmitz G. (2014). Effect of high versus low doses of fat and vitamin A dietary supplementation on fatty acid composition of phospholipids in mice. *Genes Nutr.* 9 368 10.1007/s12263-013-0368-0 PMC3896631 24306959

[B66] Wietrzych M. Meziane H. Sutter A. Ghyselinck N. Chapman P. F. Chambon P. (2005). Working memory deficits in retinoid X receptor gamma-deficient mice. *Learn. Mem.* 12 318–326. 10.1101/lm.89805 15897255 PMC1142461

[B67] Wietrzych-Schindler M. Szyszka-Niagolov M. Ohta K. Endo Y. Perez E. de Lera A. R. (2011). Retinoid X receptor gamma is implicated in docosahexaenoic acid modulation of despair behaviors and working memory in mice. *Biol. Psychiatry* 69 788–794. 10.1016/j.biopsych.2010.12.017 21334601

[B68] Wu A. Ying Z. Gomez-Pinilla F. (2011). The salutary effects of DHA dietary supplementation on cognition, neuroplasticity, and membrane homeostasis after brain trauma. *J. Neurotrauma* 28 2113–2122. 10.1089/neu.2011.1872 21851229 PMC3191367

[B69] Xia Z. Storm D. R. (2012). Role of signal transduction crosstalk between adenylyl cyclase and MAP kinase in hippocampus-dependent memory. *Learn. Mem.* 19 369–374. 10.1101/lm.027128.112 22904367 PMC3418765

[B70] Zolfaghari R. Ross A. C. (2003). Recent advances in molecular cloning of fatty acid desaturase genes and the regulation of their expression by dietary vitamin A and retinoic acid. *Prostaglandins Leukot. Essent. Fatty Acids* 68 171–179. 10.1016/S0952-3278(02)00267-3 12538081

